# P-2318. Practices Surrounding Peri-Transplant Prevention of Coccidioidomycosis: Preliminary Report from a Survey of California Transplant Centers

**DOI:** 10.1093/ofid/ofae631.2470

**Published:** 2025-01-29

**Authors:** Catherine DeVoe, Monica Fung, Hannah H Nam, Alan Koff, Cathy Logan, Sindhu Chandran, Sandy Chang, George Sakoulas, Joanna M Schaenman, Brian S Schwartz

**Affiliations:** University of California, San Francisco, San Francisco, California; UCSF, San Fransisco, California; University of Irvine - California, Orange, California; UC Davis School of Medicine; University of California, San Diego, San Diego, California; Cedar-Sinai Medical Center, Los Angeles, California; Loma Linda University, Loma Linda, California; University of California San Diego School of Medicine, San Diego, CA; University of California Los Angeles, David Geffen School of Medicine, Los Angeles, California; University of California, San Francisco, San Francisco, California

## Abstract

**Background:**

Coccidioidomycosis is a significant cause of morbidity and mortality in solid organ transplant (SOT) recipients. Although existing guidelines address some aspects of pre- and post-transplant testing, prophylaxis, and monitoring, in many cases best practices are unknown, particularly for regions with variable Coccidioides endemicity such as California. This study aims to understand existing coccidioidomycosis prevention strategies at California transplant centers.

**Figure:**
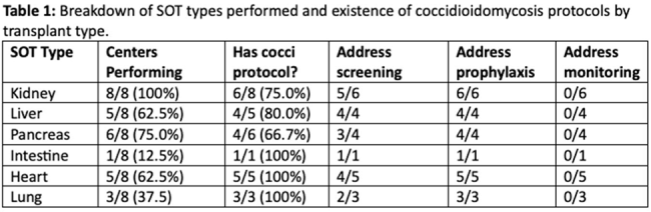

**Methods:**

We designed a web-based survey that inquired about California transplant centers’ protocols for pre-transplant coccidioidomycosis testing, post-transplant prophylaxis, and post-transplant monitoring. The Organ Procurement and Transplantation Network website was used to identify adult transplant centers in California that performed at least one SOT in 2023. Primarily pediatric centers were excluded. A transplant infectious disease or transplant physician from each center was invited to complete the survey on behalf of their institution.

**Figure:**
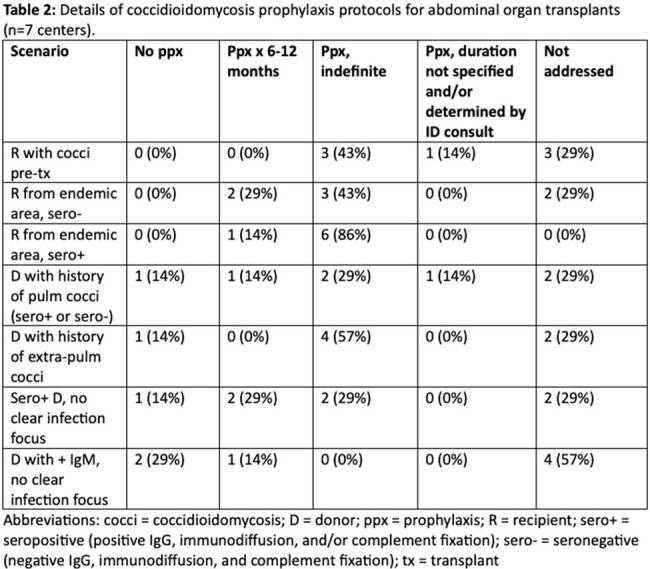

**Results:**

Eight out of 15 California transplant centers, representing 68.9% of the total SOT volume for 2023, completed the survey by the time of this report. Types of SOTs performed by the centers are shown in Table 1. All centers had at least one coccidioidomycosis protocol. Many centers had protocols that applied to multiple SOT types; no center had different protocols for the different abdominal transplant types. Details of the seven prophylaxis protocols for abdominal transplants are provided in Table 2. No center recommended Coccidioides-directed prophylaxis for all recipients.

**Conclusion:**

All transplant centers that responded to the survey had at least one coccidioidomycosis protocol, but not all had protocols that applied to all transplant types, and the amount of detail provided by the protocols varied widely. All centers provided some guidance about prophylaxis for recipients from endemic areas, and the majority recommended indefinite prophylaxis for those who were seropositive pre-transplant. Fewer centers provided guidance addressing recipients’ personal history of coccidioidomycosis or donor status. Further research is needed to clarify best practices for reducing coccidioidomycosis after SOT so that approaches can be better standardized.

**Disclosures:**

Catherine DeVoe, MD, AN2 Therapeutics: Grant/Research Support Alan Koff, MBBS, Astra Zeneca: Clinical trial support to institution Joanna M. Schaenman, MD, PhD, FAST, Eurofins Viracor: Honoraria|F2G: Grant/Research Support|MedCure: Advisor/Consultant|Moderna: Clinical trial support to institution|OneLegacy: Advisor/Consultant

